# Method of spatial scanning of modulated laser radiation for outline imaging of interphalangeal joints

**DOI:** 10.1117/1.JBO.31.7.076001

**Published:** 2026-07-01

**Authors:** Svetlana Shmavonyan, Aleksandr Khanbekyan, Marina Movsisyan, Valery Tuchin, Aram Papoyan

**Affiliations:** a Institute for Physical Research of National Academy of Sciences of Armenia, Ashtarak-2, Armenia; b Saratov State University, Institute of Physics and Science Medical Center, Saratov, Russian Federation; c Institute of Precision Mechanics and Control, FRC “Saratov Scientific Centre of the RAS,” Saratov, Russian Federation

**Keywords:** optical imaging, spatial scanning, ballistic and snake light, modulated laser, optical clearing

## Abstract

**Significance:**

Optical transmission imaging is an inexpensive and noninvasive method of examining biological tissues and structures, but due to strong scattering, its applicability is limited to millimeter-thick samples, thus preventing *in vivo* studies of centimeter-thick biological organs.

**Aim:**

The development of a technique that overcomes the limitation imposed by scattering will significantly expand the scope of the application of transillumination imaging, allowing the examination or diagnosis of biological objects of centimeter thickness, in particular, interphalangeal joints.

**Approach:**

The approach used is based on a previously developed method of highly sensitive registration of predominantly ballistic and snake photons when scanning modulated laser radiation. In addition, to obtain a contour image, a technique of selective registration across the profile of a Gaussian laser beam was used, which made it possible to obtain high spatial resolution with a large diameter of a collimated laser beam. An optical clearing agent was used to mitigate the scattering imposed by the skin.

**Results:**

The method was tested on a model object and applied to obtain the contour of the interphalangeal joint. The resulting joint contour matches the actual shape, but its width in the image is larger than the actual width. Calculations show that the size of the structure in the image also depends on the intensity and diameter of the laser beam, as well as the extinction coefficient of the sample. It is shown that, given the other parameters, the extinction coefficient can be determined from the joint image space. The effect of the clearing agent on the clarity and contrast of the image is quantitatively analyzed, and a reduction in the scattering coefficient (by 9% to 14%) is estimated.

**Conclusions:**

The suggested method has been shown to be efficient for deep-tissue imaging of biological objects up to several centimeters thick, particularly joints, revealing the outline of structural features. We have shown that the use of an optical clearing agent allows reproducible images to be obtained with noticeably higher contrast and clarity, sufficient for preliminary express diagnostics of pathologies.

## 1 Introduction

Among the most common pathologies today are various diseases of the finger joints caused by infectious, inflammatory (including rheumatoid), traumatic, degenerative-dystrophic, neoplastic, and other processes. For the early diagnosis of these diseases—particularly of arthritis—informative, noninvasive joint imaging is essential. In addition to conventional imaging methods such as radiography (including X-ray tomography), ultrasound, and magnetic resonance imaging, various optical diagnostic methods are being developed. Optical imaging is a nonionizing technique, safe for frequent use, and it does not require bulky and expensive equipment.

A Lightscan photo optical imaging technique based on transillumination of a finger joint by laser diodes with three different wavelengths and recording of a scattered light image with a charge-coupled device (CCD) camera was used by Amitai et al.^1^ for examination of a proximal interfalangeal (PIP) joint, exhibiting good agreement with ultrasonography examination. Using a similar experimental setup, a new method was proposed by Kolesnikova et al.^2^ to enhance optical imaging of human PIP joints using optical clearing of the skin. Kim et al.^3^ developed a flexible optical imaging band system to assess systemic lupus erythematosus arthritis in finger joints based on scattered light measurements at three different wavelengths. The same disease was examined by analyzing the transmitted modulated light intensity and its phase shift recorded by a CCD camera when illuminating the joints with an amplitude-modulated laser beam at frequencies 300 and 600 MHz.^4^ An overview of the appropriate optical imaging modalities for the diagnosis of rheumatoid arthritis, including bioluminescence, sagittal laser optical tomography, and photoacoustic and fluorescence imaging, was presented by Golovko et al.^5^

The effectiveness of optical imaging techniques for biomedical applications is severely limited due to strong light scattering in biological tissues, primarily in skin tissues. An effective method to mitigate the scattering problem is optical clearing based on temporary matching of refractive index in biological tissues by applying proper liquid agents, which penetrate the tissue structure (see e.g., Refs. 6–8 and the references therein). Image contrast enhancement using optical clearing was recently also used for transillumination imaging of human interphalangeal joints,^2^^,^^9^ where the change in the intensity distribution of three-wavelength illumination under the action of the optical clearing agent (a water solution of 70% glycerol and 5% dimethylsulfoxide) was analyzed using the RheumaSens medical scanner prototype.

In this paper, we propose the implementation of the earlier developed optical imaging technique based on spatial scanning of double-modulated laser radiation and sensitive selective recording of least-deflected transmitted light with infrared remote control receiver^10^ for visualization of human finger joints, assisted by optical clearing.

## 2 Experimental Arrangement and Measurement Procedure

**Fig. 1:**
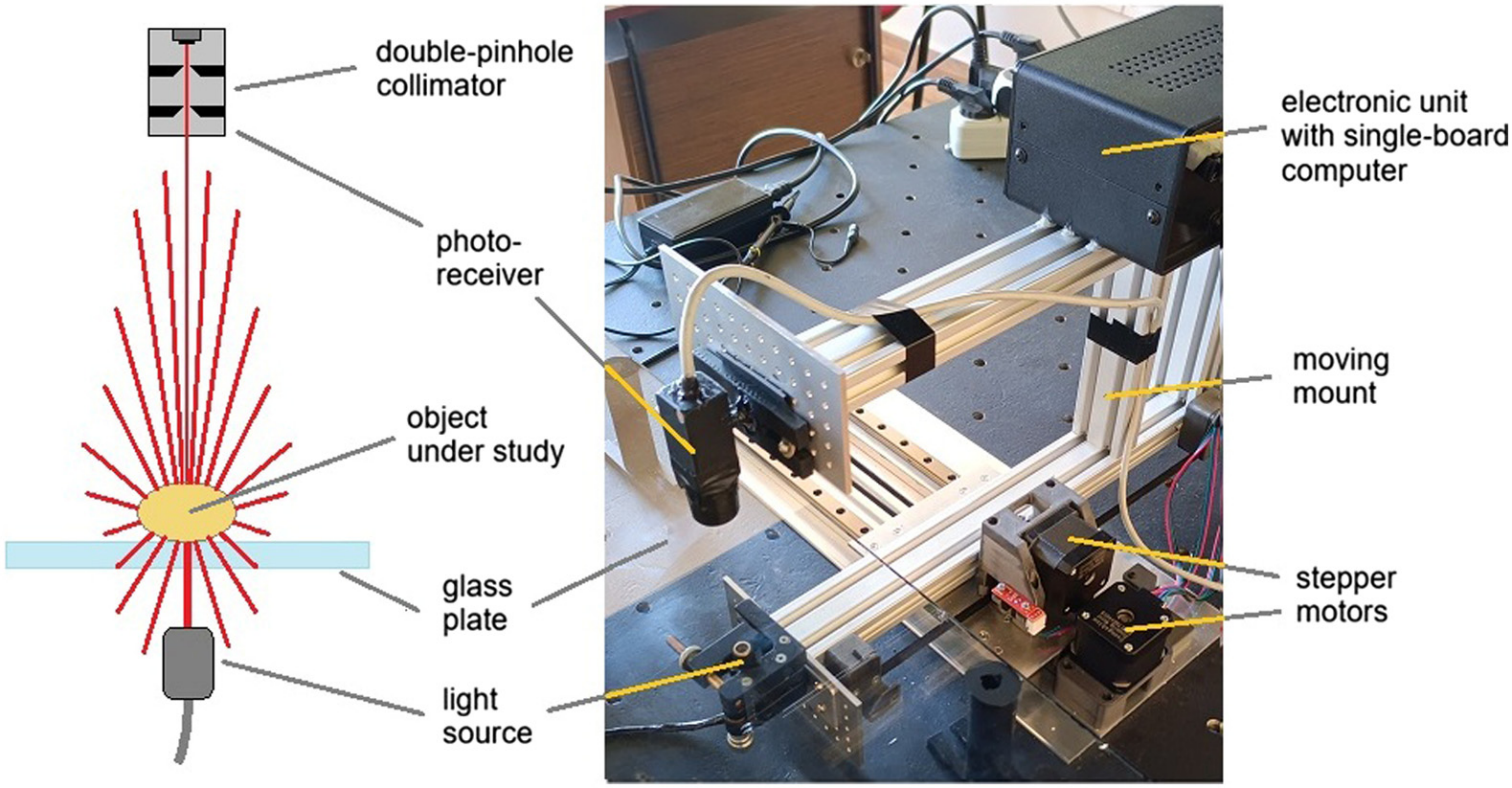
Photograph of the scanning system. Left-side drawing: schematic of the optical layout.

**Fig. 2:**
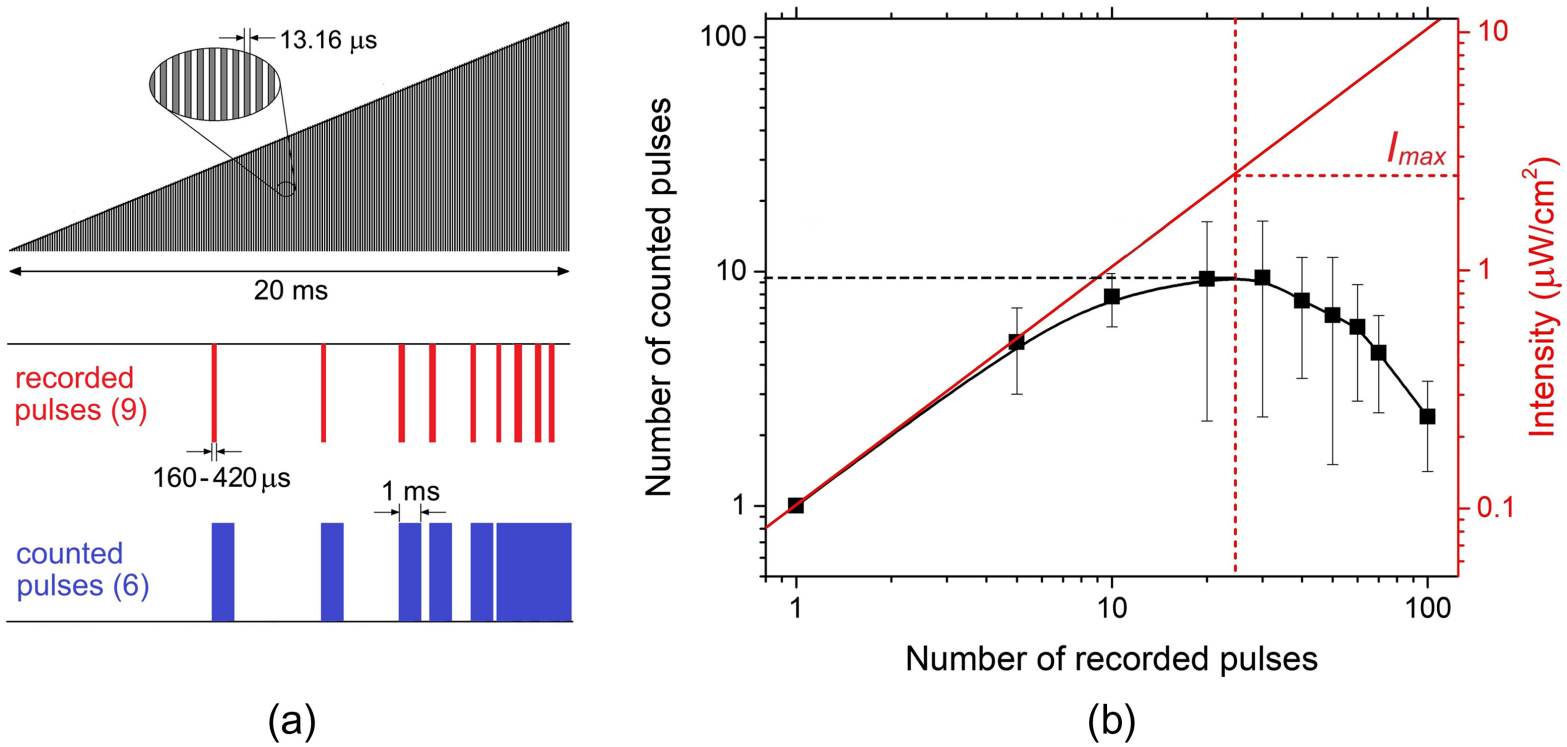
Panel (a) shows representative signal plots for laser modulation (upper diagram), photoreceiver output signal (middle diagram), and counted stretched pulses (lower diagram). Panel (b) presents a modeled dependence of the number of counted pulses on the number of photoreceiver pulses recorded on each pixel (black dots; black curve is drawn to guide the eye); red line: calibration curve of photoreceiver (dependence of the number of recorded pulses on the transmitted intensity).

The experimental setup on which the measurements were carried out is similar to that described in Ref. 10 (see the photograph in Fig. 1). A pigtail laser diode with a wavelength of 1076.3 nm and a maximum continuous-wave power of 400 mW was used as a light source. A collimator installed on the single-mode fiber termination allowed one to form a low-divergence beam of $2w_{0}=1.6\;\mathrm{mm}$ diameter (full width to the $1/e^{2}$ intensity point) at the incidence spot of the object studied. The feeding of a laser diode and amplitude modulation of laser radiation was done using a multiwave technologies cLDD laser diode controller driven by an arbitrary waveform function generator SDG5082. A 25 Hz symmetric triangular ramp envelope modulation was superimposed on a 38 kHz square carrier frequency, forming periodic radiation pulses of 20 mW average power (measured by Thorlabs, Newton, New Jersey, United States, PM100D power meter, corresponding intensity $I_{\mathrm{las}}(0)=1\;W/{\mathrm{cm}}^{2}$). The temporal sequence of the modulating pulses at each scanning step is shown in the upper diagram of Fig. 2(a).

Such a modulation algorithm is expedient for efficient recording of the light transmitted through the studied object with VS1838 receiver widely used in household electronic appliances, outright immune against any background illumination. To record radiation at a wavelength of 1076.3 nm without attenuation, a layer of colored plastic was removed from the sensor of the photoreceiver, which serves as a bandpass filter for selecting the 940 nm working wavelength used for IR remote, simultaneously forming a focusing cylindrical lens. The 2×2  mm sensor of the photoreceiver was precisely positioned in the direction of the laser beam at a distance of 11 cm from the surface of the glass plate, without using any focusing optical elements (reducing this distance to 7 cm during the measurements did not lead to a noticeable change in the recorded images). A double-pinhole collimating system consisting of two identical coaxial pinholes with Ø2  mm aperture, placed 20 mm apart from each other, was installed before the photoreceiver (see the left-side drawing in Fig. 1). This assembly, blackened from inside by a carbon nanoparticle layer, allowed the diminishing of the contribution of stray light from nearby areas of the illuminated target in the recorded signal. With this measurement configuration, with a very limited detection angle (≈11  mrad), the photoreceiver predominantly records a small number of photons, which experience the least deviation when propagating through a highly scattering medium, thus allowing the formation of low-distortion images. A similar geometric selection has previously been used for spatially resolved measurements of ballistic transmission in tissue samples.^11^

To obtain an image of the object under study, it was placed on an unmovable glass plate between a light source (laser fiber collimator) and a photoreceiver, mounted opposite each other on a Π-shaped two-axis (X and Y) translation stage, which was scanned across the object area, driven by stepper motors. Scanning was performed with the same 0.19 mm step in the X and Y directions at a speed of 50 steps (pixel pitches) per second. The measurement time for each pixel was 20 ms, during which time, thanks to the triangular envelope modulation of laser radiation with a frequency of 25 Hz, the intensity of the incident light changes linearly from zero to the maximum value [see the upper diagram in Fig. 2(a)]. Output (low-level) signal is formed when the photoreceiver records a burst of at least six consecutive pulses of the 38 kHz carrier modulation. A typical width of the output signal is 160 to 420  μs, as is shown in the middle diagram of Fig. 2(a). The conversion circuit at the photoreceiver output forms inverted and stretched pulses with a fixed duration of 1 ms, which are counted and recorded for each pixel by the Python program to form an output dataset and a grayscale image.

It is important to note that with these recording parameters, a maximum of 20 signal pulses will be recorded on each pixel. For the larger number of signal pulses (higher light intensity), they will overlap, thus reducing the number of counted events (those having leading edges, independently of duration). This is illustrated in the lower diagram of Fig. 2(a). Such a pulse stretching procedure allows attenuating intense components in transmitted light, thus equalizing the dynamic range of visualized image for efficient revealing of uniform structural features such as vascular or bone structure, independently of the strongly varying overall transmission level in samples with nonuniform thickness.

Within a single pixel, the recorded pulses arrive at random moments in time, and for the same number of pulses during a pixel time (20 ms), the number of counted stretched pulses can vary significantly. To simulate the order of a given number of incoming pulses, we used a random number generator producing a given number (from 1 to 100) of short pulses recorded by the photoreceiver at arbitrary moments within the 20 ms period, running it 10 times to improve the prediction accuracy. Stretching of these pulses to 1 ms results in partial overlap, thus reducing the number of counted pulses. Figure 2 shows the obtained dependence of the number of counted pulses on the number of pulsed recorded by the photoreceiver. One can see that for the given duration of stretched pulse (1 ms), the strongest response (9 to 10 counted pulses) is expected for 20 to 30 recorded pulses, corresponding to $I_{\max}\approx 2.5\;\mu W/{\mathrm{cm}}^{2}$ of the transmitted intensity (measured value, see dashed lines). At the same time, the number of recorded (short) pulses, at least in the range of up to 100 events, shows a linear dependence on the power of the transmitted radiation (see the red calibration curve in Fig. 2).

It should be noted that the dependence of the number of counted pulses on the number of registered pulses shown in Fig. 2 is obtained by simulating a random sequence of detected events. The actual dependence may differ slightly at each pixel depending on the specific sequence of the finite (and small) number of detected pulses. To form an outline (contour) image, which is intrinsically nonlinear, a pronounced maximum must be present in the middle part of this dependence. Its position and width depend on the duration of the stretched pulses, which can to some extent affect the contrast and clarity of the resulting image. This maximum forms a “registration window” corresponding to a certain range of transmitted intensity. The maximum will not be formed both for a very low level of transmitted light (no detected pulses) and for a high light level (completely overlapped pulses, which will be detected as a single pulse). Application of a linear modulation of laser radiation power from zero to the maximum amplitude on each pixel makes the registration window sliding (floating), assuring automatic adjustment of a needed counting mode within the modulation period.

The laser beam at the output of the fiber collimator has a Gaussian spatial profile. Its intensity at a distance r from the center is described by:

$$I_{\mathrm{las}}(r)=I_{\mathrm{las}}(0)\exp(\frac{-2r^{2}}{w_{0}^{2}}),$$ (1)

where $I_{\mathrm{las}}(0)$ is the intensity of the radiation in the center of the beam, and $w_{0}=0.8\;\mathrm{mm}$ is the radius of the beam (half width to the intensity point $1/e^{2}$). The beam diameter in the present experiment is 8.4 times larger than the scanning step, and the intensity distribution at each pixel can be considered flat-top. Note that the diameter of the incident beam can be reduced by focusing, but this is impractical for the centimeter-thick samples that are being studied.

**Fig. 3:**
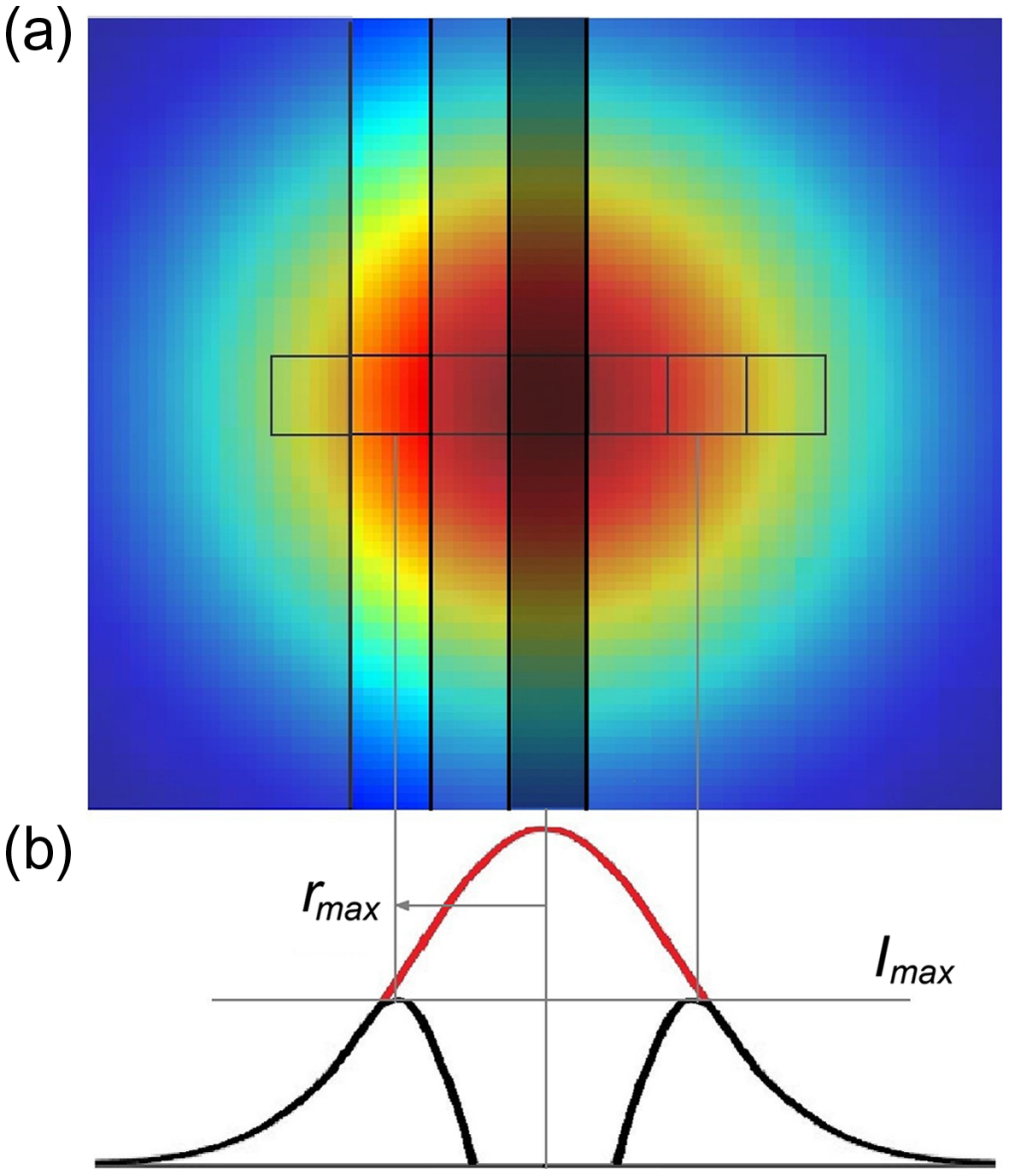
(a) Gaussian profile of the laser beam; squares indicate the scanning steps in one direction. (b) Transmitted intensity distribution across the profile (red curve) and distribution of counted pulses (black curve). A slit placed at a distance $r_{\max}$ from the beam center, where the transmitted radiation intensity is close to $I_{\max}$ (see Fig. 2), produces a much brighter image than a slit at the center.

With this measurement configuration and pulse counting scheme, contribution to the outline image of the structural feature of the studied sample is provided when the laser beam is slightly away from it (within the diameter of the laser beam). This is illustrated in Fig. 3.

**Fig. 4:**
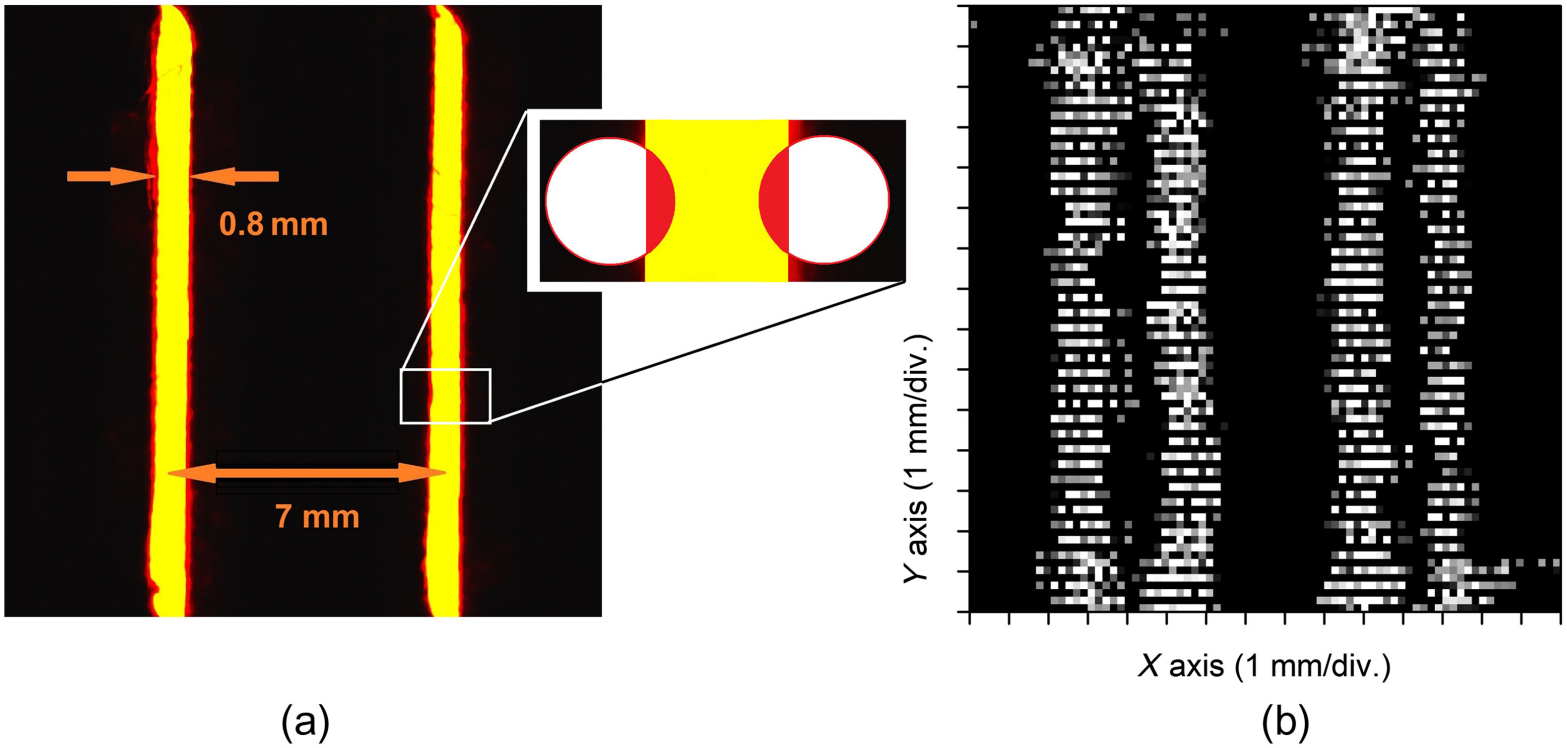
(a) Opening slits in nontransparent craft paper used in a model object. (b) Transilluminated image of the model object sandwiched between the seven layers of 1 mm-thick polyethylene foam packaging film [the same scaling is applied for panels (a) and (b)]. The inset schematically shows the positions of the laser beam forming the bright strips.

To verify the above-described imaging mode, the scanning system has been tested on a model object composed of a sheet of dense craft paper with a=0.8  mm opening slits [Fig. 4(a)], sandwiched between strongly scattering polyethylene packaging film layers of 1 mm thickness (four layers from the top and three layers from the bottom). The recorded grayscale image is shown in Fig. 4(b), which exhibits a three-strip (white-black-white) structure of the slit.

The appearance of such a structure is consistent with the schematic explanation given in Fig. 2. At a distance $r_{\max}$ from the edge of the slit, the level of light intensity is close to $I_{\max}$, producing a bright strip (see the inset in Fig. 4); but approaching the central part of the slit, the level of light becomes high enough to overlap individual stretched pulses, thus reducing the count to zero (a black strip).

As can be seen in Fig. 4(b), the distance between the neighboring slits in the image is the same as for the model object (7 mm), whereas the slit width, measured as the mean distance b between the white strips applying a five-point window adjacent averaging procedure, is $b=a+2r_{\max}=2.1\;\mathrm{mm}$, from which we find $r_{\max}=0.65\;\mathrm{mm}$. It should be noted that this procedure for determining the $r_{\max}$ is approximate, not taking into account the continuous nature of light diffusion in the scattering medium.

Let us calculate under what conditions this $r_{\max}$ value is obtained. The intensity of radiation transmitted through an absorbing and scattering medium is determined by the Beer–Lambert–Bouguer law:

$$I_{\mathrm{tr}}(r)=I_{\mathrm{las}}(r)\exp(-\mu d),$$ (2)

where d is the distance (sample thickness), and μ is the extinction coefficient, which comprises contributions from scattering and absorption.

Combining Eqs. (1) and (2), we obtain an expression for $r_{\max}$:

$$r_{\max}=\sqrt{\frac{w_{0}^{2}}{2}(\ln\frac{I_{\mathrm{las}}(0)}{I_{\max}}-\mu d)}.$$ (3)

By this equation, substituting the known values of the parameters for the case of Fig. 4: $r_{\max}=0.065\;\mathrm{cm}$, $w_{0}=0.08\;\mathrm{cm}$, $I_{\max}=2.5\;\mu W/{\mathrm{cm}}^{2}$, $I_{\mathrm{las}}(0)=1\;W/{\mathrm{cm}}^{2}$, and d=0.7  cm, we can calculate the only unknown value, the extinction coefficient μ. The value obtained is $\mu =16.5\;{\mathrm{cm}}^{-1}$. Despite the large variation in the values of the extinction coefficient for low-density polyethylene foam films depending on the pore size, this value associated with strong scattering is consistent with the available data.^12^

As can be seen from Eq. (3), the value of $r_{\max}$ and hence the spacing of the recorded pattern are affected by the intensity of the incident laser beam, the scattering and absorbing properties of the sample, and $I_{\max}$, which is dependent on the duration of stretched pulses.

## 3 Joint Imaging: Results and Discussion

Subsequent measurements were performed on the interphalangeal joints of human fingers (hand of one of the co-authors, AP, age 66 years). Before starting the measurements, the irradiation conditions were checked to ensure that they comply with the requirements of the ANSI Z136.3 standard for safe use of lasers in healthcare. The measured average laser power in the modulation mode used in this study was 20 mW with a beam diameter of 1.6 mm, corresponding to an intensity of $1\;W/{\mathrm{cm}}^{2}$. Considering that the exposure time at each scanning step (0.2 mm) is 20 ms and the beam diameter is 8.4 times greater than the scanning step, the laser exposure time on each skin area is 170 ms, corresponding to an exposure of $0.17\;J/{\mathrm{cm}}^{2}$. This value is much below the maximum permissible exposure of $3.31\;J/{\mathrm{cm}}^{2}$ set by ANSI Z136.3 for a wavelength of 1076.3 nm and a duration of exposure of 170 ms.

**Fig. 5:**
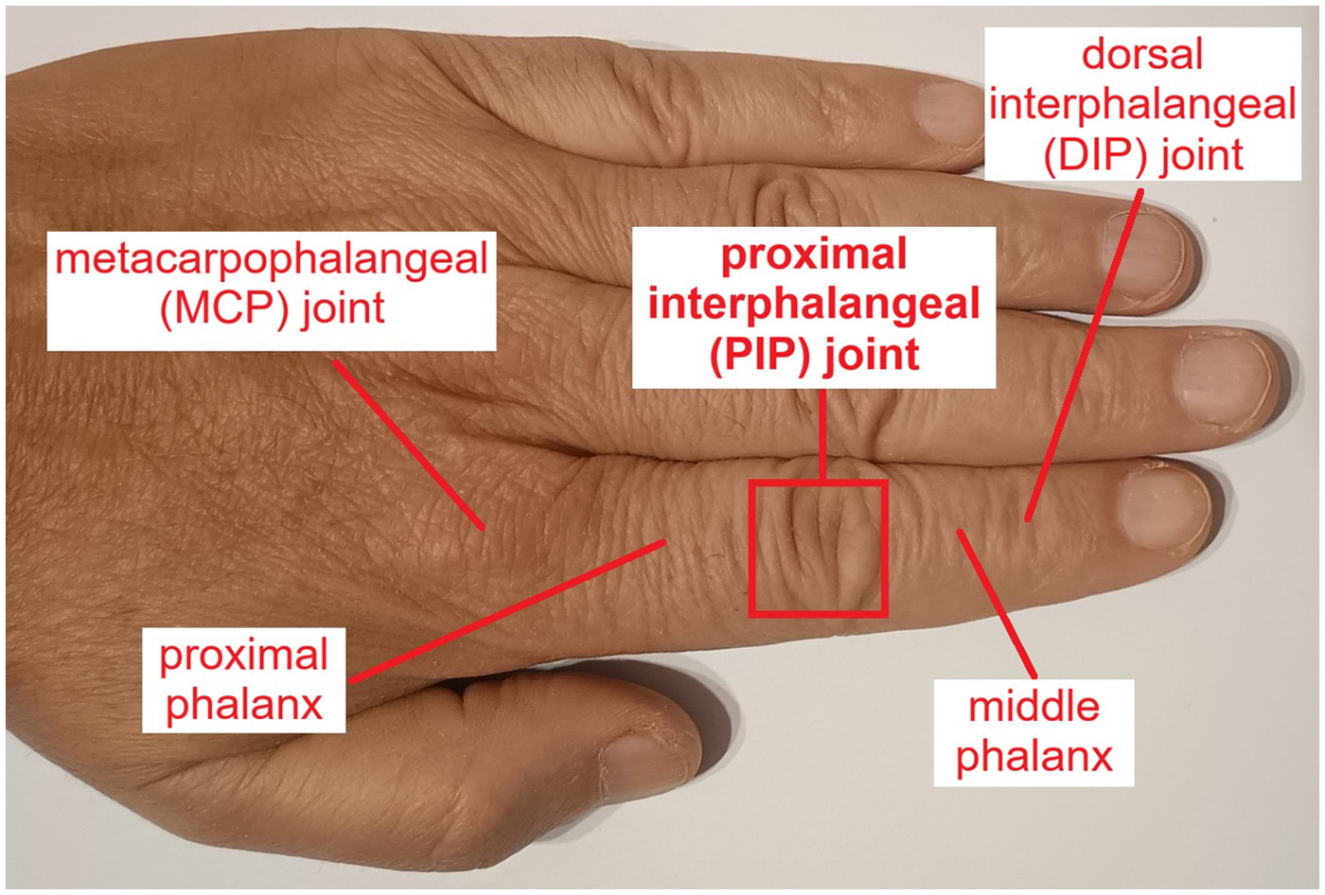
Photograph of the scanned human hand. Red square shows 15×15  mm scanned area of the proximal interphalangeal (PIP) joint of the index finger.

Scanning was carried out on an area of 15×15  mm with a step of 0.19 mm (80×80  pixels, scanning time 2 min). In this recording mode, images of all dorsal and proximal interphalangeal joints (DIP and PIP), as well as metacarpophalangeal joints, were reliably recorded. Regular measurements were taken for the d=21  mm-thick proximal interphalangeal joint of the index finger of the left hand (Fig. 5).

**Fig. 6:**
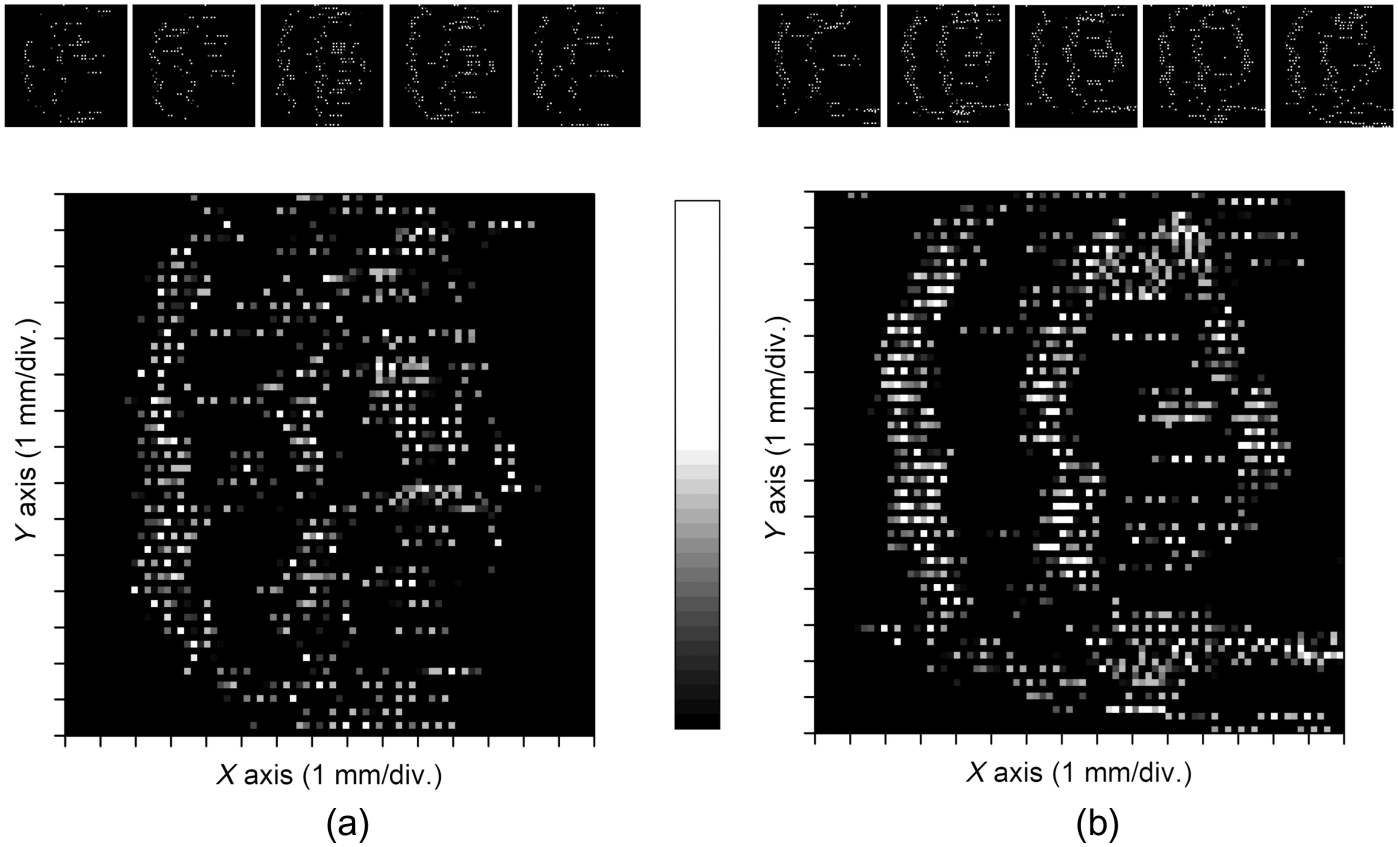
Upper row: five consecutively registered raw images of a motionless PIP joint; lower panels: pixel-wise summed patterns of the top row images: (a) before applying a clearing agent; (b) 40 min after applying a clearing agent.

The images obtained revealed the structure of the joint, but the clarity and contrast were inferior to those of the model object. Moreover, the reproducibility of the results during repeated scanning for a fixed joint position was insufficient [see the upper row of images of Fig. 6(a)]. Although these imperfections can be caused by the system-related effects, such as essentially nonlinear detection response, limited efficiency of ballistic photon collection for deep tissue, detection geometry, and sensitivity to alignment, it can nevertheless be assumed that the main reason is the strong scattering on the skin (the skin is known to contribute the most to the scattering^13^).

To test this supposition, a second series of measurements was performed using an optical clearing agent. A mixture of glycerol (85%), propylene glycol (10%), and dimethyl sulfoxide (5%) was used as a clearing agent. To ensure sufficient penetration of the skin with the clearing agent, measurements were carried out 30 to 40 min after the application of the composition (five consecutive scans were performed in 10 min while keeping the joint in invariable position). The imaging results for the same joint with the clearing agent applied are shown in Fig. 6(b). To evaluate the reproducibility of the imaging results, the lower panels of this figure represent summed patterns by pixels of the respective top row images.

**Fig. 7:**
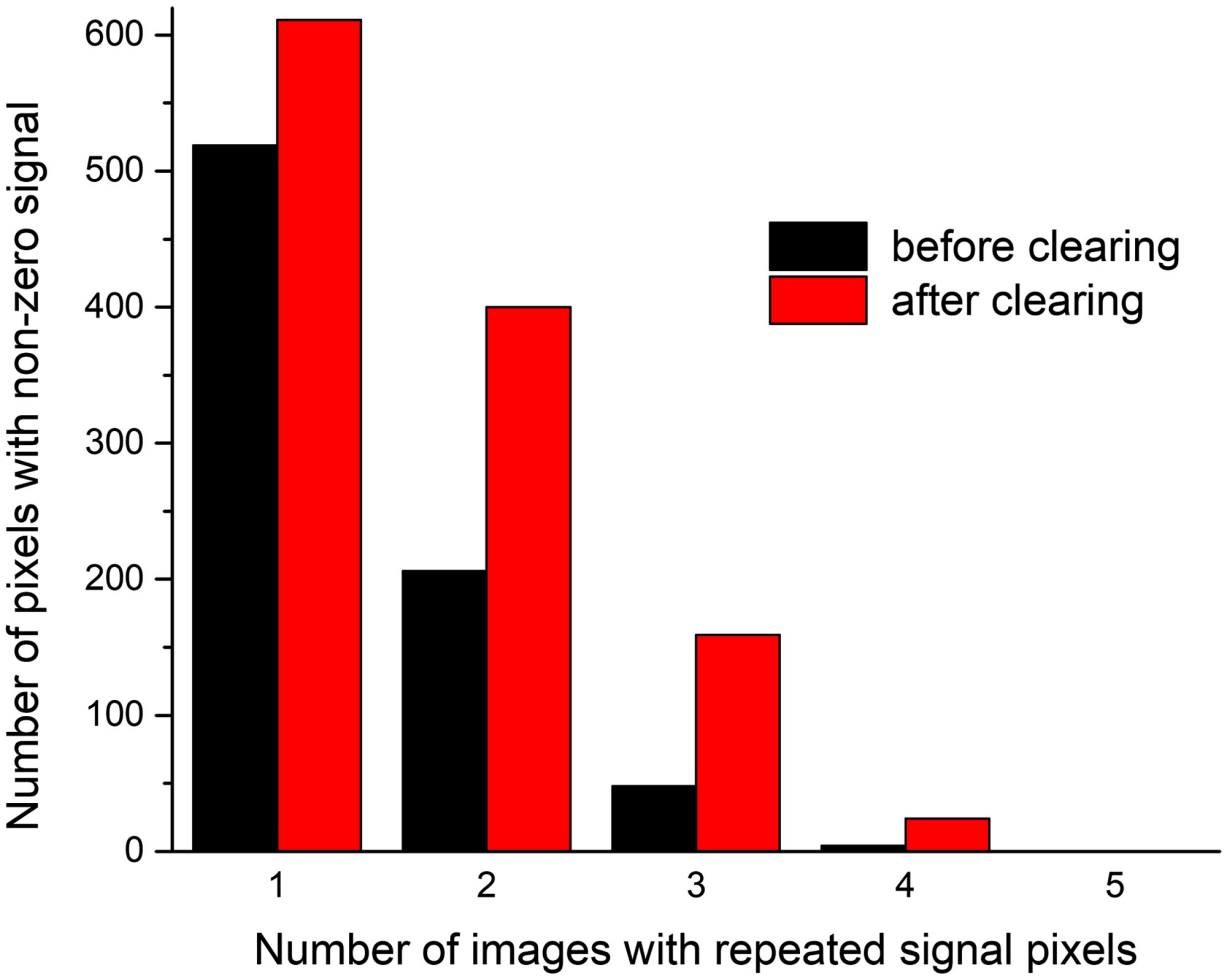
Reproducibility diagram of five images before and after clearing. The Y-axis represents the number of pixels with a nonzero signal that are not repeated in the other four images (X=1) and are repeated in two, three, and four images out of five (X=2,3,4).

Obviously, the optical clearing contributed to the improvement in the clarity and reproducibility of the images. The following procedure was implemented to quantify the influence of optical clearing. For each of the five recorded images before and after clearing, we have analyzed reproducibility of nonzero signals for each of the particular pixels. The result of this analysis is presented in Fig. 7. As seen in the diagram, the clearing results in a noticeable increase in the number of coinciding signal pixels (1.9, 3.3, and 6.0 times for coincidences in two, three, and four images, respectively). On the other hand, optical clearing leads to an increase in the total number of pixels with a nonzero signal (by 1.54 times), as well as the total intensity of the registered signal (by 1.22 times).

In Fig. 6(b), the structure of the synovial cavity located between the articular cartilages of the PIP joint is clearly visible as a dark vertical curved strip outlined with light strips spaced approximately b=3.8  mm apart. The corresponding value before optical clearing [Fig. 6(a)] is on average 5% lower (b=3.6  mm). Note that this result is not consistent with the result reported in Ref. 14, where treatment of skin with an optical clearing agent leads to a substantial decrease in the diameter of the blood vessel in the recorded image compared with the case of turbid skin. This contradiction is explained by the significant difference in the measurement methods.

Let us now analyze the results obtained. Unlike in the case of the model object, here, we have two unknown parameters, the extinction coefficient μ and the joint space a. Taking into account that $r_{\max}=(b-a)/2$, we can derive from Eq. (3) an expression for the extinction coefficient:

$$\mu =\mu_{s}^{\prime }+\mu_{a}=\frac{1}{d}(\ln\frac{I_{\mathrm{las}}(0)}{I_{\max}}-\frac{(b-a{)}^{2}}{2w_{0}^{2}}),$$ (4)

where $\mu_{a}$ is the absorption coefficient, and $\mu_{s}^{\prime }=\mu_{s}(1-g)$ is the reduced scattering coefficient characterizing light transport in turbid media by combining the scattering coefficient $\mu_{s}$ and the anisotropy factor g=⟨cos θ⟩, which represents the equivalent isotropic scattering coefficient, accounting for forward-directed scattering (high g).

Taking for a the extreme limits of the width of the healthy PIP joint space for males, 0.9 to 1.3 mm (see Ref. 15 and the references therein) and substituting the known values $w_{0}=0.08\;\mathrm{cm}$, d=2.1  cm, $I_{\max}=2.5\;\mu W/{\mathrm{cm}}^{2}$, $I_{\mathrm{las}}(0)=1\;W/{\mathrm{cm}}^{2}$, and b=0.36  mm (0.38 mm) before (after) optical clearing, respectively, we obtain μ=3.43 to $4.17\;{\mathrm{cm}}^{-1}$ before optical clearing, and μ=3.01 to $3.82\;{\mathrm{cm}}^{-1}$ after optical clearing. This 9% to 14% reduction in the extinction coefficient should be attributed to the decrease in $\mu_{s}^{\prime }$ caused by reduced scattering of the skin.

The values of the interphalangeal joint extinction (absorption and scattering) coefficients for near-infrared available in the literature vary greatly (orders of magnitude), depending on the object of measurement (synovial fluid, synovial tissues, cartilages, skin contribution), methodology, wavelength, pathologies, etc. The values obtained for μ in our work agree, in particular, with the result of Atwal et al.,^16^ where the average effective attenuation coefficient for healthy PIP joints was found to be $4.6\;{\mathrm{cm}}^{-1}$. However, it should be noted that Eq. (4) cannot be considered exact because the measured width b depends on both the response of the system and the properties of the tissue, and consequently, the obtained extinction coefficient value is approximate.

**Fig. 8:**
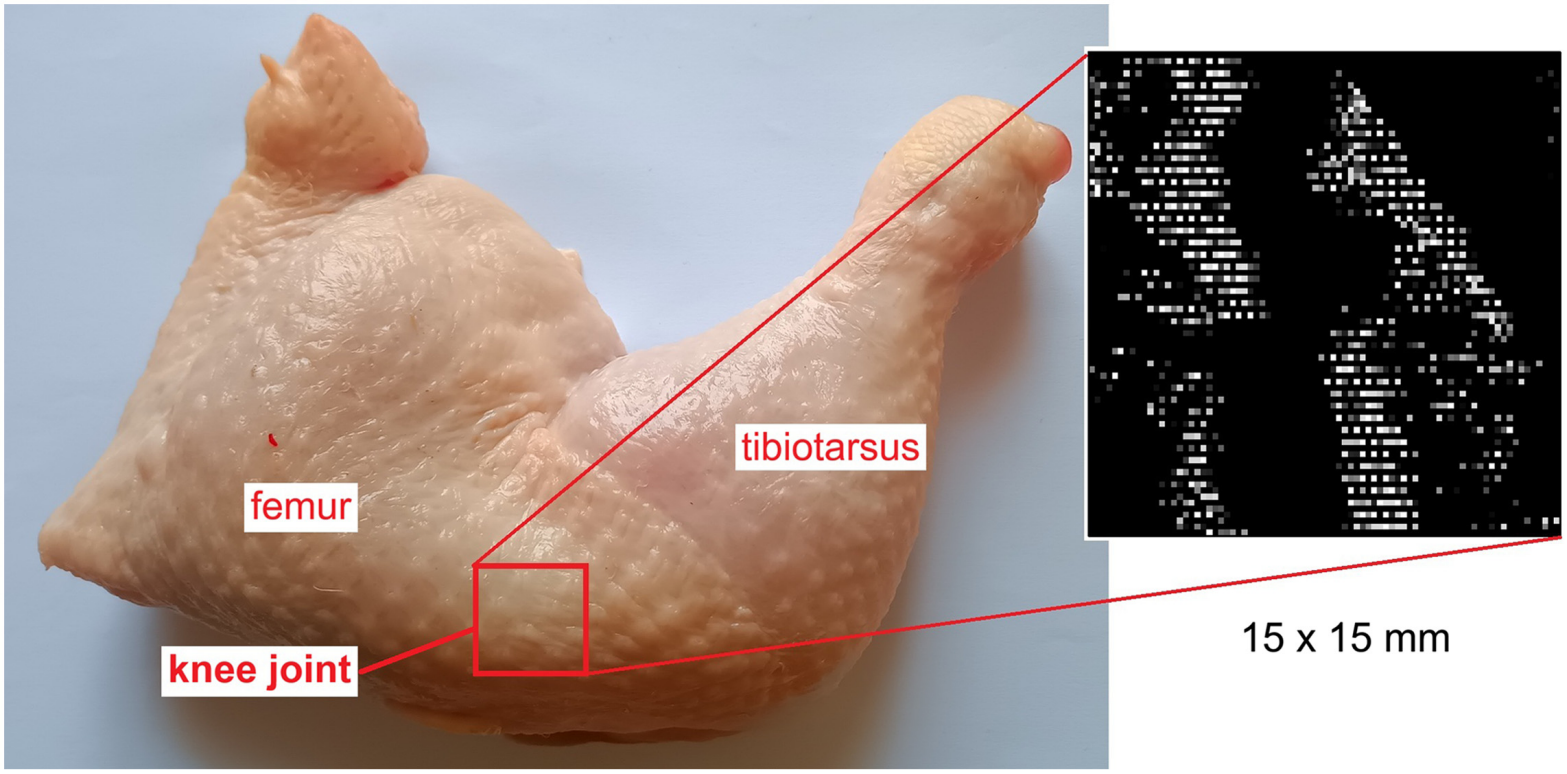
40-mm-thick chicken leg (thigh and drumstick). The red square marks the scanned area of the knee joint. The inset shows the recorded image.

To check the applicability of the method for greater sample thickness, a knee joint of a chicken leg (see Fig. 8) was chosen as the next object of study. Despite its greater overall thickness (around 40 mm in the joint area), the sizes of chicken femur and tibiotarsus bones linked with a knee joint are close to the sizes of phalanx bones of the human PIP joint. The measurement was done without applying an optical clearing agent, keeping all the modulation and scanning parameters invariable.

The recorded 80×80  pixel image covering the 15×15  mm joint area marked by a red square is shown in the inset of Fig. 8. The obtained image is sufficiently clear and high contrast and is perfectly reproducible when rescanned. The image reveals a dark vertical region with an average width of 3.3 mm and a sharp curvature in the center. This dark region is surrounded by light regions spaced ∼5.5  mm apart. This structure represents the synovial cavity filled with transparent synovial fluid, which is located between the articular cartilages attached to the bones of the femur and tibia. The observed fracture of the gap, in its central part, may be associated with the cruciate ligament, which connects the bones through the synovial cavity. It can be concluded that the interphalangeal joint space of the chicken joint is somewhat larger than in the case of the human PIP joint (Fig. 6). However, quantitative size comparison based on the measurements performed with the proposed method is questionable because of the significant differences between the objects being compared. The images allow to compare the shapes of the interphalangeal gaps, but not their sizes.

The fact that the clarity of the outline image of a 40-mm sample is no worse than in the case of a 20-mm sample supports a significant contribution of skin in overall scattering. A fresh, featherless chicken thigh transmits light better than a human palm or fingers. This is due to the much thinner skin of the chicken, its high moisture content, and the simple structure (two layers versus three layers of human). It should also be noted that the recording of outline images with comparable contrast and clarity for biological objects with significantly different thicknesses indicates the effectiveness of linear envelope modulation of laser radiation power, which allows for automatic adjustment of the “sliding window” of registration as the thickness changes.

## 4 Conclusion

The main goal of this study was to test the feasibility of a spatial scanning system with an amplitude-modulated laser beam and sensitive selective pulsed recording of the transmitted signal for joint structure imaging. It is demonstrated that, in spite of the large diameter of the collimated laser beam, comparable with the interphalangeal joint space, this technique allows visualizing the shape of the joint (more precisely, the interphalangeal space with synovial fluid) but distorts its real size. However, it can be useful for revealing foci of inflammation, cartilage and ligament destruction, deposits of uric acid crystals, structural deformations, and other pathologies, which will affect the sharpness, shape, and spacing of the recorded images.

Our further work will focus on revealing other possible factors contributing to the image spreading, such as lensing (defocusing) action of the curved structure of the joint, waveguide effects in articular cartilage and synovial fluid, etc. We plan to optimize the scanning, modulation, and recording parameters, as well as optical clearing conditions, for a more accurate retrieval of the actual dimensions of the joint space width. We also plan to refine the technique by adding a circular angular scanning unit, which will allow recording a tomographic image of the joints, and to extend the method for imaging of vascular and tendon structures.

## Data Availability

Data underlying the results presented in this article are not publicly available at this time but may be obtained from the authors upon reasonable request.
